# Conversion of Lignocellulosic Biomass to Nanocellulose: Structure and Chemical Process

**DOI:** 10.1155/2014/631013

**Published:** 2014-08-27

**Authors:** H. V. Lee, S. B. A. Hamid, S. K. Zain

**Affiliations:** Nanotechnology & Catalysis Research Centre (NANOCAT), 3rd Floor, Block A, Institute of Postgraduate Studies (IPS), University of Malaya, 50603 Kuala Lumpur, Malaysia

## Abstract

Lignocellulosic biomass is a complex biopolymer that is primary composed of cellulose, hemicellulose, and lignin. The presence of cellulose in biomass is able to depolymerise into nanodimension biomaterial, with exceptional mechanical properties for biocomposites, pharmaceutical carriers, and electronic substrate's application. However, the entangled biomass ultrastructure consists of inherent properties, such as strong lignin layers, low cellulose accessibility to chemicals, and high cellulose crystallinity, which inhibit the digestibility of the biomass for cellulose extraction. This situation offers both challenges and promises for the biomass biorefinery development to utilize the cellulose from lignocellulosic biomass. Thus, multistep biorefinery processes are necessary to ensure the deconstruction of noncellulosic content in lignocellulosic biomass, while maintaining cellulose product for further hydrolysis into nanocellulose material. In this review, we discuss the molecular structure basis for biomass recalcitrance, reengineering process of lignocellulosic biomass into nanocellulose via chemical, and novel catalytic approaches. Furthermore, review on catalyst design to overcome key barriers regarding the natural resistance of biomass will be presented herein.

## 1. Introduction

Owing to the overconsuming of petroleum resources and increasing demand of fossil-based fuels and chemical, it is necessary to develop renewable resources to produce biofuels and biochemical for economical and sustainable development. Lignocellulosic biomass industry has become green, possible alternative of fossil resources in order to compensate the increasing trend of world's demand for petroleum usage [1]. This type of biomass is the most abundantly available biopolymer in nature. It is estimated that the worldwide production of lignocellulosic biomass is about 1.3 × 10^10^ metric tons per annum [2, 3]. The lignocellulosic resources included (i) agricultural residues (palm trunk and empty fruit bunch, corncobs, wheat straw, sugarcane bagasse, corn stover, coconut husks, wheat rice, and empty fruit bunches); (ii) forest residues (hardwood and softwood); (iii) energy crops (switch grass); (iv) food wastes; and (v) municipal and industrial wastes (waste paper and demolition wood) [4, 5]. The high availability of biomass has appeared to be one of the most potential resources of transportation fuels and chemicals platform. Transformation of cheaper biomass into value-added product by the mean of converting “carbon source” into “carbon sink” indicates that carbon can be fully utilized before it would be released into the atmosphere [6–8]. Reconstruction of low cost lignocellulosic materials to products with superior functions presents a feasible option for improvement of energy security and greenhouse emissions reduction. With the availability of biomass, it is believed that this technology is capable of turning negative cost of biomass (plant waste) into positive-earning materials.

Lignocellulose is a complex carbohydrate polymer, containing polysaccharides built from sugar monomers (xylose and glucose) and lignin, a highly aromatic material. Lignocellulosic biomass fractionation into reactive intermediates, such as glucose, cellulose, hemicellulose, and lignin, is a critical process prior to further development into liquid fuels, chemicals, and other end products. The lignocellulosic biomass consists of defensive inner structure which has contributed to the hydrolytic stability and structural robustness of the plant cell walls and its resistance to microbial degradation. On the other hand, the presence of cross-link between cellulose and hemicellulose with lignin via ester and ether linkages [9–11] leads to the biomass recalcitrance. Thus, it is important to understand the chemistry of biomass in order to deconstruct the material into component that can be chemically or catalytically converted into biomass-derived fuels, chemicals, or reactive intermediate.

This paper provides an overview of lignocellulosic biomass reengineering into nanocellulose reactive intermediate by discussing (i) biomass recalcitrance, (ii) chemical approaches for lignocellulosic biomass fractionation, (iii) nanocellulose synthesis via chemical route, and (iv) new prospects of solid catalyst for nanocellulose synthesis. Finally, conclusions on the catalyst development for targeted cellulosic nanomaterial products from lignocellulosic biomass deconstruction will be drawn based on some literature study.

## 2. Overview of Refinery of Lignocellulosic Biomass into Nanocellulose

Global focus is currently directed towards lignocellulosic biomass valorization which not only is limited to liquid biofuel and chemicals production [12–14] but also involves synthesis of reactive intermediate (nanocellulose) for further end-product processing [15]. Nanocellulose, which is obtained from cellulose, is creating a revolution in biobased materials for diverse applications. The nanocellulose (cellulose nanofibers) is equipped with various superior characteristics which are nanoscale dimension, high surface area, unique optical properties, high crystallinity, and stiffness (comparable to Kevlar and steel) together with the biodegradability and renewability of cellulose. This has made this intermediate a precious green alternative to materials, construction, packaging, automobile, transportation, and biomedical fields [15–18].

**Figure 1:**
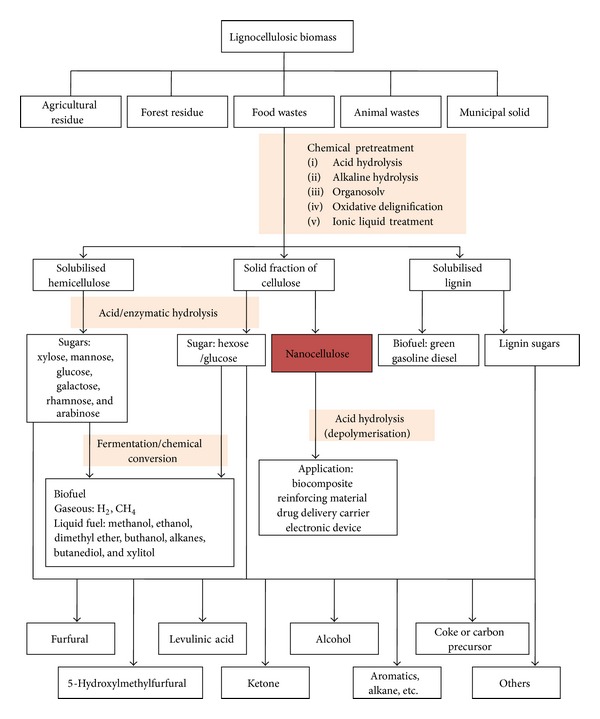
Roadmap of lignocellulosic biomass biorefinery to nanocellulose intermediate and chemicals.

Lignocellulosic biomass consists mainly of three biopolymers: (i) cellulose (~30–50% by weight), (ii) hemicellulose (~19–45% by weight), and (iii) lignin (~15–35% by weight) [2, 4, 19]. These polysaccharides are associated with each other in a heteromatrix to different degrees and varying composition depending on the type of biomass, species of plant, and even source of the biomass [9]. The chemical composition of biomass for different types of agriculture, industrial, and forestry wastes is shown in Table 1 [20, 21]. The relative abundance of cellulose, hemicellulose, and lignin is the key factor in determining the feedstock suitability for nanocellulose production. Generally, a biomass pretreatment step is necessary to ensure the separation of cellulose component from tight bond of polymeric constituents (cellulose, hemicellulose, and lignin) in lignocellulosic biomass. The main intention of this fractionation treatment is to increase the accessibility of cellulose fiber to chemical attack prior to mild hydrolysis of isolated cellulose, by cleaving the ether bond between glucose chain in order to produce nanosize cellulose intermediate [22, 23]. However, biomass fractionation is a very complex process as high recovery of polysaccharides (cellulose, hemicellulose, and lignin) is required so that all three components can be fully converted into useful end products. Sometime, the biomass pretreatment shall lead to over depolymerisation of polysaccharide chains and subsequent sugar ring opening, which produce undesirable product such as glucose, acid, alcohol, and aldehyde [24]. But such separation is mandatory step to unlock the stored fiber for effective utilization of nanocellulose in the current nanotechnology field. Chemical treatments are the most popular pretreatment technologies in the isolation of cellulose fibers to nanocellulose. An overall roadmap for lignocellulosic biomass reengineering to nanocellulose intermediate and chemical platform is shown in Figure 1. The conversion included the pretreatment of biomass to separate cellulose from noncellulosic contents and further refinery of cellulose, hemicellulose, and lignin fraction for valuable products.

## 3. Lignocellulosic Biomass Recalcitrance

**Figure 2:**
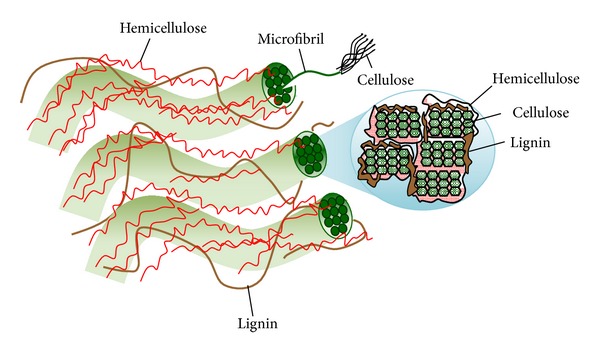
Plant cell wall structure and microfibril cross-section (strands of cellulose molecules embedded in a matrix of hemicellulose and lignin).

Lignocellulose is the primary building block of plant cell walls. The complex hierarchy structure of lignocellulosic biomass is the main obstacle for key components fractionation, where cellulose, hemicellulose, and lignin are hindered by many physicochemical, structural, and compositional factors.  Figure 2 showed the plant cell wall structure and different biocompositions. Generally, cellulose fibrils are coated with hemicellulose to form an open network, whose empty spaces are gradually filled up with lignin [66–68]. Several Factors contributed to biomass recalcitrance are (i) high lignin content; (ii) protection of cellulose by lignin; (iii) cellulose sheathing by hemicellulose; (iv) high crystallinity and degree of polymerization of cellulose; (v) low accessible surface area of cellulose; and (vi) strong fiber strength. Thus, the degree of cellulose isolation with high recovery of hemicellulose and lignin compounds is key for a given biomass fractionation [69, 70].

**Figure 3:**
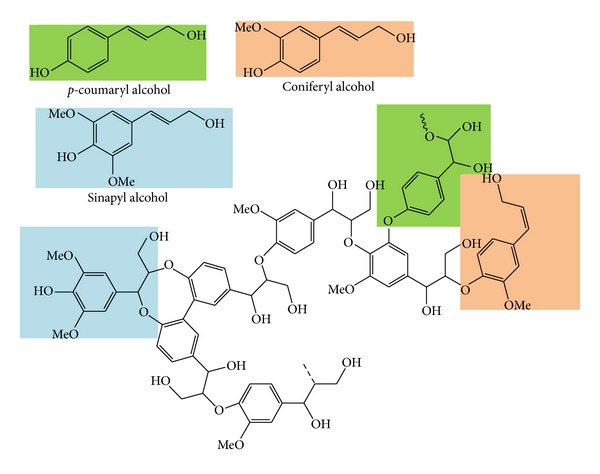
Chemical structures of lignin (*p*-coumaryl alcohol, coniferyl alcohol, and sinapyl alcohol).

Lignin is a complex molecular structure containing cross-linked polymers of phenolic monomers especially* p*-coumaryl alcohol, coniferyl alcohol, and sinapyl alcohol (Figure 3). The presence of lignin in lignocellulosic biomass is the main obstacle of biomass recalcitrance during separation process. Lignin act as a protective barrier for plant cell permeability and resistance against microbial attacks and thus prevents plant cell destruction. Basically, softwood consists of higher amount of lignin compared to other types of biomass; it makes softwood more recalcitrant and resistant than the other feedstock in cellulose separation step. As a result, removal of lignin is necessary to enhance biomass digestibility up to the point where both hemicellulose and cellulose are exposed for solubilisation process [71, 72].

**Figure 4:**
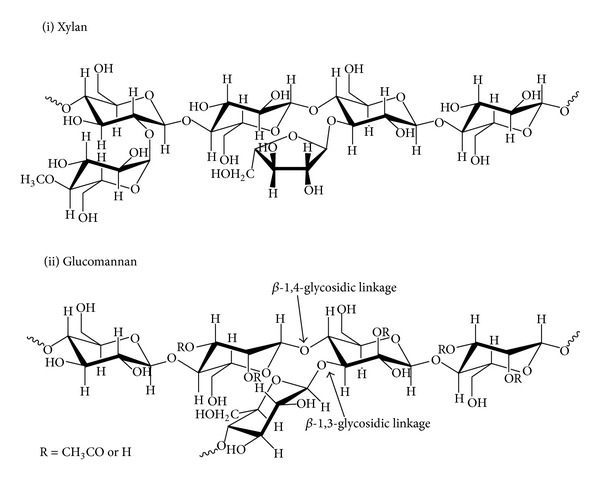
Chemical structure of hemicellulose compounds (xylan and glucomannan are the most existing biopolymer).

Other than lignin, the accessibility of cellulose is affected by obstructions caused by hemicellulose. Generally, the cellulose fibrils are “coated” with hemicellulose branches with short lateral chains consisting of different sugars (pentoses, hexoses, and acetylated sugars) (Figure 4). It has been suggested that a minimum of 50% of hemicellulose should be removed to extensively increase cellulose digestibility [70]. Comparing to cellulose, hemicellulose can be easily hydrolysed by diluted acid, alkali, or enzymes under mild conditions. Due to its high thermochemical sensitivity, hemicellulose degradation can easily occur to form unwanted coproducts (furfurals and hydroxymethyl furfurals), which inhibits the fermentation process for bioethanol production. However, it does not affect nanocellulose synthesis [70, 73]. For this reason, pretreatment severity is usually a compromise to maximize lignin and hemicellulose recovery during separation process while maintaining cellulose structure for further nanocellulose synthesis.

Different lignocellulosic biomass pretreatment strategies are currently available with variation in terms of pH, temperature, types of catalyst, and treatment time. These variations affect the severity of the pretreatment and the biomass composition during biomass degradation [21]. Several types of pretreatment that are used to open biomatrix structures are categorized into the following: (i) physical (milling and grinding); (ii) chemical (alkaline, dilute acid, oxidizing agents, and organic solvent); (iii) biological; and (iv) multiple or combinatorial pretreatment of physical and chemical techniques (steam pretreatment/auto hydrolysis, hydrothermolysis, and wet oxidation) [5, 20, 24, 69, 70, 74–77]. Among the biomass pretreatment, chemical pretreatment proved to be the most efficient method and cost effective for biomass deconstruction with low pretreatment severity. Physical pretreatment includes chipping, grinding, and milling and thermal methods are less efficient and consume more energy than chemical methods, while enzyme for biological pretreatment is expensive and it takes longer pretreatment duration [21]. The detailed chemical pretreatment for lignocellulosic biomass will be further discussed in Section 4.

**Figure 5:**
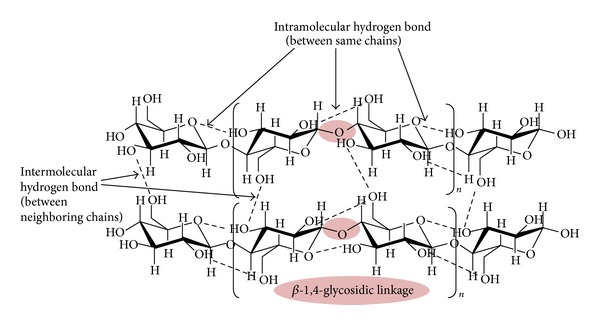
Chemical structures of cellulose chains.

Cellulose in biomass present in both crystalline and amorphous forms is found in an organized fibrous structure. The long chain cellulose polymers consist of* D*-glucose subunits which are linked together by *β*-1,4 glycosidic bonds. These linear polymers are linked together by different inter- and intramolecular bonds, which allow them to be packed side by side in planar sheet and bundled into microfibrils (Figure 5). Hence, the cellulose is insoluble in water as the hydroxyl groups in sugar chains are bonded to each other, making a hydrophobic scenario. For this reason, the crystalline domain of microfibrils cellulose, with the presence of extensive intermolecular hydrogen bond and Van Der Waals force makes it another challenge for hydrolysis accessibility to nanocellulose synthesis [78]. In this chemical system, only the cellulosic chains exposed on the surface of the microfibril are easily accessible to solvents, reactants, and chemicals. Thus, the reactivity of cellulose toward hydrolysis is very low.

To improve the performance of cellulose depolymerisation for nanocellulose synthesis, the supramolecular structure of cellulose should be disrupted; that is, some crystalline domains should be converted into amorphous phases. For this purpose, several types of hydrolysis process by chemical or catalytic route have been extensively explored (acid hydrolysis, alkaline hydrolysis, delignification via oxidation, organosolv pretreatment, and ionic liquids pretreatment). Chemistry of depolymerisation of cellulose into nanodimension by acid hydrolysis is another recent interest of study which will be further discussed in Sections 5 and 6.

## 4. Fractionation of Lignocellulosic Biomass via Chemical Pretreatment

The recalcitrance (resistance of plant cell walls to deconstruction) of lignocellulosic biomass is a major obstacle in the separation of cellulose, hemicellulose, and lignin for different application. The recalcitrance is due to the highly crystalline structure of cellulose which is embedded in a matrix of polymers-lignin and hemicellulose. The main goal of pretreatment is to overcome the recalcitrance which is targeted to alter the size and structure of biomass thru separation of cellulose from the matrix polymers and create access for hydrolysis to turn cellulose into nanocellulose with controllable reaction. Generally, an ideal fractionation of cellulose from lignocellulosic biomass should meet the following requirements: (i) avoid the structure disruption or loss of cellulose, hemicellulose, and lignin content; (ii) be cost effective and reduce energy input; and (iii) minimize production of toxic and hazardous wastes.

**Figure 6:**
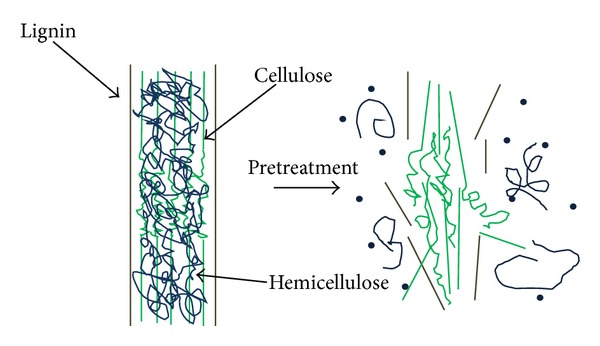
Deconstruction of lignocelluloses into cellulose, hemicellulose, and lignin.

The biomass pretreatment is aimed to break the lignocellulosic complex, solubilise the noncellulosic contents (lignin and hemicellulose) but preserve the materials for further valorization, reduce cellulose crystallinity, and increase the porosity of the materials for subsequent depolymerisation process [79, 80] (Figure 6). The present chemical treatments for lignocellulosic biomass degradation are (i) acid hydrolysis, (ii) alkaline hydrolysis, (iii) oxidation agent, (iv) organosolv, and (v) ionic liquids.  Table 2 showed operation profiles and degree of fractionation for chemical pretreatments on lignocellulosic biomass degradation. Different types of chemical pretreatment render selective functionality in biomass degradation. Some of the chemical selectively solubilise hemicellulose whilst some chemicals solubilise lignin components. However, all these chemical treatments effectively remove and recover most of the hemicellulose portions as soluble sugars in aqueous solution. The functionalities, advantages, and limitation of different pretreatment are summarized in Table 3. By pointing out that the main barrier in biomass fractionation is the complexity of plant cell structure, it is suggested that the list of chemical pretreatment can be applied individually or in a combination of techniques using several treatment steps to achieve high yield of (nano)cellulose with high recovery of hemicellulose and lignin under low energy input and low severity of process.

*(i) Acid Hydrolysis.* Acid pretreatment is a process to break the rigid structure of lignocellulosic material in which hydronium ions breakdown and attack intermolecular and intramolecular bonds among cellulose, hemicellulose, and lignin in biomass hierarchy structure. The acid hydrolysis includes concentrated and dilute acid solutions where different levels of acid severity contribute to various biomass fractionated products [69]. Concentrated acids such as H_2_SO_4_, HCl, H_3_PO_4,_ and HNO_3_ are being used to hydrolyse biomass [28, 33]. It is an effective agent for biomass deconstruction to maximize the yield of monomeric sugars for biofuel production. In nanocellulose synthesis, long chain structure of cellulose feedstock is preferable for further reaction to produce nanocellulose. The concentrated acid used in acid hydrolysis is toxic, hazardous, and corrosive; thus highly corrosion resistant reactor and extreme care in handling are required in the process. This makes acid pretreatment an expensive option. In addition, the concentrated acid must be recovered after pretreatment to make the process economically and environmentally feasible. As a result, the effects for different type of acid, pH, reaction temperature, and reaction time in biomass fractionation process are crucial to influence the maximum yield of targeted cellulose product. Extreme reaction condition will cause uncontrollable degradation of biomass to sugars and subsequently catalyzed the degradation of monosaccharide to undesirable coproducts such as acid and alcohol [21, 79].

For the reasons stated above, dilute-acid hydrolysis has become cost-effective alternative to enhance biomass separation to isolate cellulose, hemicellulose, and lignin which can further be used for nanocellulose synthesis and chemical production. Generally, dilute acid (e.g., concentration of H_2_SO_4_ < 4 wt.%) [26, 27] can dissolve and recover most of the hemicellulose as dissolved sugars up to 100% conversion under low process severity (low temperature and low acid concentration). The remaining fraction of lignin in the residue cellulose from hemicellulose solubilisation process will then be removed via cellulose purification process. Cellulose purification is a process that utilizes alkaline pretreatment, where delignification occurs to separate lignin from cellulose in order to increase accessibility of cellulose for hydrolysis reaction.

*(ii) Alkaline Hydrolysis.* The major strategy of alkaline pretreatment is to disrupt the lignin structure in biomass, thus improving the susceptibility of the remaining polysaccharides (cellulose and hemicellulose) for other treatment [74]. Alkaline hydrolysis occurs at milder conditions (below 140°C) and lower severity as compared to other pretreatment technologies. The mechanism involves saponification of intermolecular ester bond, which crosslinks xylan (hemicellulose) and lignin [74]. The porosity of the pretreated material will increase with the removal of the crosslinker. Cleavage of this ester linkages are substituted by nucleophilic acyl in the presence of alkaline salt (e.g., NaOH or Ca(OH)_2_) to form a carboxylic salt and an alcohol [21]. Furthermore, the alkaline degradation of lignin also involved cleavage of two types of aryl ether bonds: C_aliphatic_–O–C_aromatic_ and C_aromatic_–O–C_aromatic_, which produces acids (ferulic acid and* p*-coumaric acid). Generally, the pretreatment agents that are used for alkaline hydrolysis are NaOH, KOH, Ca(OH)_2_, hydrazine, and ammonium hydroxide [30, 31]. In the presence of alkali, it also serves the following functions: (i) swelling agent to cellulose; (ii) leading to an increase of internal surface area; (iii) leading to a decrease in the degree of polymerization and crystallinity of cellulose; (iv) leading to partial solvation of hemicellulose; (v) destroying the structural linkages between lignin and carbohydrate by saponification of intermolecular ester bonds; and (vi) disrupting the lignin structure by breaking its glycosidic ether bond. Lignin will fail to act as protective shield to the cellulose after lignin solubilisation step, thus making extracted cellulose more susceptible to nanosynthesis.

These steps are needed to avoid over degradation of cellulose to sugar; it is suggested that alkali pretreatment is implemented after dilute acid treatment to remove hemicellulose component from lignocellulosic material. During hemicellulose solubilisation, there is a strong layer of lignin keeping the cellulose unexposed/inaccessible, which led to less degradation of cellulose to glucose. This process shall maximize the cellulose yield for nanocellulose synthesis. The challenge to complete the biomass delignification is difficult as this component is normally located within the deep cell wall, hydrophobic, physically stiff, strong poly-ring bonds of C–O–C, C–C, and tendency to recondensation [81]. Thus, the remaining cellulose and part of lignin will be subjected for further separation process to solubilise lignin matrix while maintaining a high recovery rate of low crystalline cellulose for nanocellulose production.

*(iii) Oxidation Agent.* Oxidation agent such as organic peroxide (H_2_O_2_, C_2_H_4_O_3_), ozone, oxygen, or air is another technique used to catalyze delignification process by attacking and cleaving of lignin's ring structure [34, 35]. Normally, oxidation agents are used to enhance the effects of alkaline pretreatments. Under basic condition of pH > 12, the oxygen will reduced to superoxide radical (−O_2_^•^), where the ring will open by this nucleophilic attack [81]. It is an efficient treatment to oxidize aromatic ring of lignin and part of hemicellulose polymer to carboxylic acids compounds (e.g., formic acid, oxalic acid, and acetic acid). This treatment is suitable for extraction of cellulose as the oxidation agent is more aggressive on lignin and partially on hemicellulose, while cellulose is hardly decomposed under this mild condition [82].

*(iv) Organosolv.* Organosolv pretreatment is the simultaneous process of lignin and hemicellulose degradation, solvation, and solubilisation of lignin fragments from lignocellulosic feedstock with the presence of organic solvents or their aqueous solutions. Organic solvent acts as a dissolving agent by solubilising lignin and some of the hemicellulose under heating condition and leaving a relatively pure solid cellulose residue. The common solvents used for organosolv pretreatment are low boiling point alcohol such as methanol, ethanol, acetone, ethylene glycol, and ethyl acetate [83]. The OH^−^ ion from alcohol solvent will attack the acid-ester bonds of lignin-hemicellulose compounds. The cleavages of ether linkages from lignin and minor hydrolysis of glycosidic bond in hemicellulose are important for the breakdown of aromatics and polysaccharides of lignocellulose.

An advantage of employing high volatility alcohol is due to the ease of recovery by simple distillation which requires very low energy consumption. In the other hand, these alcohols are lower in cost and soluble in water. Addition of catalyst in organosolv pretreatment such as inorganic (HCl or H_2_SO_4_) or organic acid (oxalic, acetylsalicylic and salicylic acid) helps to break the internal bonding of lignin-hemicellulose linkage and improve the organosolvation process under lower temperature. Consequently, this pretreatment involve simultaneous prehydrolysis and delignification of lignocellulosic biomass to solubilise noncellulosic components and obtain cellulose fraction. Furthermore, presence of organic solvent was also found to swell the cellulose and reduce crystallinity of cellulose for further application [83, 84].

At present day, the organosolv pretreatment is not economically feasible to be utilized. Extensive washing is needed to wash the pretreated materials with organic solvent prior to water washing in order to avoid the precipitation of dissolved lignin, which leads to cumbersome washing arrangements. Furthermore, recovery of organic solvents causes increase of energy consumption for the whole process [85].

*(v) Ionic Liquids.* Ionic liquids (ILs) pretreatment is another recent development in chemical-based dissolution pretreatment technology. The tunability of ILs chemistry makes it highly capable of dissolving wide variety of biomass type. Unlike the heterogeneous reaction environment (cellulose in water), ILs makes the catalytic sites highly access the *β*-glycosidic bonds, which facilitates the reaction of biomass fractionation and hydrolysis of cellulose [86, 87]. ILs can dissolve cellulose, hemicellulose, and lignin under considerably mild conditions without degrading the chain's structure. It is reusable liquid salts at room temperature, typically composed of inorganic anion and organic cation, which can be tuned to generate different dissolving capacity for targeted components. The common examples of these ILs include salts of organic cations for cellulose dissolution and biomass pretreatment, such as 1-alkyl-3-methylimidazolium [C_*n*_mim]^+^; 1-alkyl-2,3-dimethylimidazolium [C_*n*_mmim]^+^; 1-allyl-3-methylimidazolium [Amim]^+^; 1-allyl-2,3-dimethylimidazolium[Ammim]^+^; 1-butyl-3-methylpyridinium [C_4_mP_*y*_]^+^; and tetrabutylphosphonium [Bu4P]^+^ with *n* = number of carbons in the alkyl chain [88, 89].

An ideal ILs for lignocellulosic biomass pretreatment process should possess the following: (i) high dissolution capacity for different component by varying organic cation; (ii) low melting point; (iii) low viscosity; (iv) low/no toxicity; and (v) high stability. Considering the fact that fractionation of cellulose from noncellulosic matrix is a complicated process, chemistry of ILs is (anion and cation compositions) needed to be adjusted in order to solubilise hemicellulose and lignin, to ease cellulose fractionation from the biomatrix [90, 91]. For example, 1-ethyl-3-methylimidazolium acetate [Eminm]Ac has high solubility for lignin and low solubility for cellulose. It is able to selectively extract the rigid lignin from the lignocellulose while yielding a highly amorphous cellulose fraction [78, 84].

Other than biomass degradation to isolate cellulose product, ILs pretreatment could reduce crystallinity of cellulose to amorphous nature. Cellulose in the form of fibrils poorly hydrolyse or depolymerise under mild condition due to its intermolecular hydrogen linkages between polysaccharide chains. ILs is capable of disrupting the hydrogen bonds by forming another hydrogen bond between anion of IL with cellulose (sugar hydroxyl protons) in a 1 : 1 ratio. This will break up the cellulose hydrogen bonded structure, thus decreasing the compactness of cellulose and making it more amorphous and susceptible to depolymerisation process [92].

In summary, the chemical pretreatment for fractionation of lignin and carbohydrates (cellulose and hemicellulose) can be achieved through the use of acids, alkalies, solvents, and ionic liquids, which promote selective solubilisation for each biocomponent (Table 3). The presence of acid is responsible for solubilising hemicellulose and cellulose via hydrolysis reaction; thus controllable acid concentration is crucial to promise high recovery of cellulose fibers for further action. Lignin is not hydrolysed by acid, but it can be soluble in alkali treatment and oxidized in the presence of oxidation agent, while organosolv treatment mainly focuses on solubilisation of carbohydrates. Ionic liquid can dissolve both carbohydrates and lignin, which disrupt the intricate network of noncovalent interactions between these polymers. This treatment can reduce lignin content and cellulose crystallinity.

**Figure 7:**
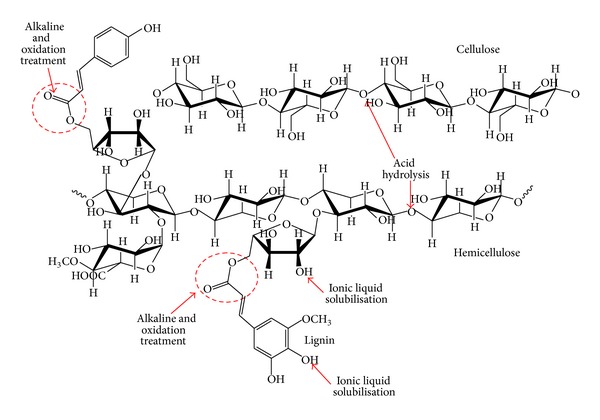
Selectivity of chemical treatments for fractionation of lignocellulosic biomass.

Although chemical process may not be as selective as biological treatment (enzyme), it consisted of many advantages related to operation period, scalability, and process control.  Figure 7 showed the chemical pretreatments on selective part of biomass compounds.

## 5. Depolymerisation of Native Cellulose to Nanocellulose

**Figure 8:**
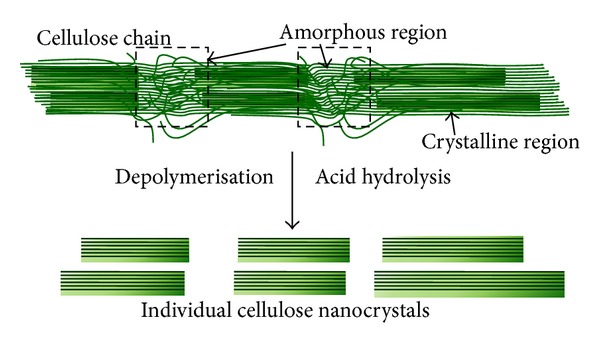
Depolymerisation of cellulose to nanocellulose.

Cellulose microfibril contains crystalline and amorphous regions that are randomly distributed along their length. The former, cellulose chain, is packed closely, whereas the latter is in disorder manner, which easily breaks under harsh treatment (Figure 8). Nanocellulose is categorized into nanocrystalline cellulose (NCC) and nanofibrillated cellulose (NFC), with extraordinary properties which was induced by nanoscale effect. Both types of nanocellulose are chemically similar; the dissimilar physical characteristic (different colloidal forms) is cellulosic “rice” and cellulosic “spaghetti” for NCC and NFC, respectively. Nanocellulose has a rigid rod-shaped structure, is 1–100 nm in diameter, and is tens to hundreds of nanometers in length. The relative degree of crystallinity and the geometrical aspect ratio (length to diameter; L/d) are very important parameters controlling the properties of nanocellulose [15, 93].

Recently, preparation of nanoscale cellulosic particles under mild conditions is getting attention from the researchers. The top-down destructuring of cellulosic fibers can be conducted by a mechanical reaction (cryocrushing, grinding, and high pressure homogenization) [46, 48, 49, 51, 94, 95], biological reaction (enzymatic treatment) [96, 97], and chemical reaction (oxidation and acid hydrolysis) [43, 45, 46, 93] (Table 4). A major obstacle which needs to be overcome for successful commercialization of nanocellulose is the high energy usage from mechanical disintegration of the fibers into nanofibers, often involving several paths through the disintegration device. Therefore, researchers have combined mechanical pretreatments with chemical techniques to increase efficiency of sizes reduction before homogenization, which helps to lower the energy consumption.

Among the cellulose depolymerisation treatments, oxidation pretreatment is one of the common techniques used to disintegrate cellulose into nanocellulose by applying 2,2,6,6-tetramethylpiperidinyl-1-oxyl (TEMPO) radicals. TEMPO-mediated oxidation method generates sinter-fibrillar repulsive forces between fibrils by modifying surface of native cellulose. This led to conversion of primary hydroxyls in cellulose into carboxylate groups, which later become negatively charged and resulted in repulsion of the nanofiber, thus contributing to an easy and fast fibrillation [94, 98].

Acid hydrolysis is a process related to the breakage of *β*-1,4 glycosidic bonds in cellulose. It is well known that acid hydrolysis is the most effective process currently to produce nanocellulose with possibility in lowering energy consumption [20, 22, 95–97, 99]. Commonly, hydrolysis is performed in the presence of mineral acid (50–72% H_2_SO_4_) for depolymerisation of cellulose. The low density of amorphous regions in native cellulose is more accessible to acid and more susceptible to hydrolytic action than the crystalline domains. These amorphous regions will break up, releasing the individual crystallites cellulose when subjected to acid treatment.

**Figure 9:**
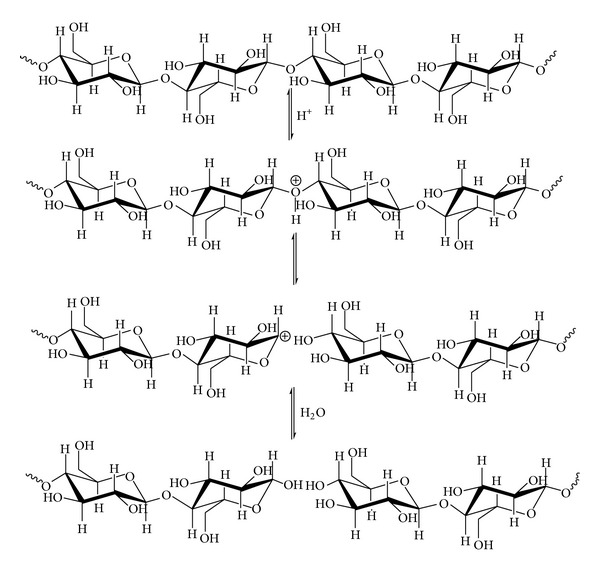
Acid hydrolysis of cellulose in to nanocellulose.

**Figure 10:**
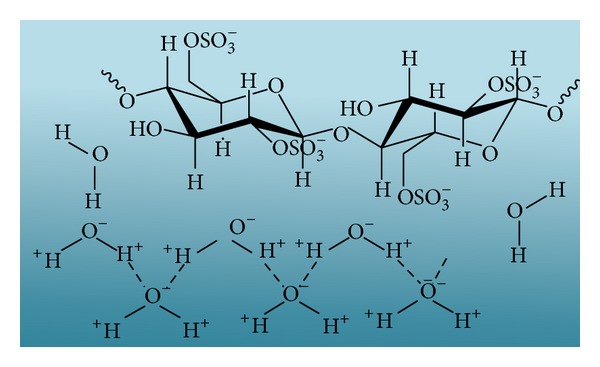
Formation of sulfate group on the nanocellulose surface after hydrolysis reaction.

The characteristic of nanocellulose from acid hydrolysis is highly dependent on various factors, such as origin of cellulose sources, types of acid, concentration of acid, reaction time, and temperature for hydrolysis. In the presence of Bronsted acidic media, the acid acts as a catalyst by protonating the oxygen atom of glycosidic bond in cellulose chain [18]. Subsequently, the unstable positive charged group leaves the polymer chain and it is replaced by the hydroxyl group of water (Figure 9). Furthermore, esterification process occurred in between H_2_SO_4_ and hydroxyl groups to yield “cellulose sulfate” which resulted in negatively charged surface of the cellulose crystallites. The anionic stabilization via the attraction/repulsion forces of electrical double layers is the reason for the stability of colloidal suspensions of crystallites (Figure 10) [97]. However, the produced nanocellulose may be chemically modified by sulfate ester group where further functionalisation of nanocellulose will be limited [100]. Besides, the conventional acid hydrolysis treatment for nanocellulose synthesis is environmentally incompatible and economically unfriendly as extra cost will be generated for effluents treatment [54].

Thus, researchers have claimed that the use of enzymatic hydrolysis may benefit from the environment point of view on comparing with acid treatment [101]. Biological-based hydrolysing agent cellulase (composed of multicomponent enzyme system) allows restrictive and selective hydrolysis of specified component in the cellulosic fibers. Enzymatic hydrolysis process involved multistep catalyzed reaction in which solid crystal of cellulose is initially disordered at the solid-liquid interface via the synergistic action of endoglucanases and exoglucanases/cellobiohydrolases. Generally, endoglucanases act to cleave the internal bonds (e.g., noncovalent interaction) present in the amorphous structure of cellulose. Besides, exoglucanases/cellobiohydrolases will further attack the terminal glycosidic bond from the end of the exposed cellulose chains generated by endoglucanases. Subsequently, short cellulose chains from initial reaction are accompanied by further hydrolysis, where beta-1,4 glycosidic linkage of cellulose is broken down by cellobiases/beta-glucosidases into nanocellulose or even glucose product.

Although enzymatic route for nanocellulose synthesis offers the potential for higher yields, higher selectivity, lower energy costs, and milder operating conditions than chemical processes, such technology was still hindered by economical (costly cellulase enzyme) and technical (rate limiting step of cellulose degradation with long processing period) obstacles. The slow rate of enzymatic hydrolysis is easily influenced by several reasons: structural features resulting from pretreatment and enzyme mechanism [102–104].  Table 5 showed the limiting factors that affect cellulase hydrolysis performance.

## 6. Future Prospect of Acid Catalyst Development for Hydrolysis of Cellulose

Acid hydrolysis can happen in homogeneous or heterogeneous catalyze reaction with first order kinetic rate. Typically, the acid hydrolysis of cellulose that uses homogeneous catalysis is more feasible in terms of mass transfer and reaction efficiency. However, these methods have major drawbacks such as (i) low chemical recovery, (ii) reactor and equipment corrosion, (iii) extra cost for waste effluents treatment, and (iv) uncontrollable selectivity of nanocellulose production. This can conclude that commercial scale of nanocellulose production by using liquid acid (e.g., H_2_SO_4_ and HCl) is not economically and environmentally sustainable.

To date, a solid acid catalyst is getting attention in selective cellulose hydrolisation. This type of acid catalyst with Bronsted acid active sites is a good alternative to concentrated H_2_SO_4_ for hydrolysis reaction. It has numerous advantages over liquid H_2_SO_4_ in terms of activity, selectivity, catalyst lifetime, and reusability. Moreover, the use of solid acid proficiently could reduce acid pollutants and cost of waste water treatment and thus reduce the manufacturing costs [99, 105–112].

The main challenge of solid catalysts is the contact between catalyst active sites with solid macromolecule of cellulose. As both reactant and catalyst are present in solid phase, it is difficult for catalytic acid sites to have a close contact with *β*-1,4 glycosidic linkage in cellulose for hydrolysis reaction. Hence, hydrothermal condition (water as reaction medium) is a good choice to improve the accessibility of catalyst to cellulose. Other than acting as mass transfer medium, water can take part as catalyst for autohydrolysis process. The hydronium ions (H_3_O^+^) formed on the surface of catalyst lead to promotion of cellulose hydrolysis.

The critical characteristics for ideal acid catalysts for hydrolysis are as follows.

- It must be a water-tolerant acid catalyst.
- It must have strong acidity and high accessibility of acid active sites for reaction.
- It shall ease in separation between solid catalyst and final nanocellulose product in the powder or gel form.

Based on these ideal criteria, several possible green acid catalysts are highly potential to be designed for cellulose hydrolysis (Table 6).

- Nanoparticle hydrolysis catalysts: they disperse in water solution capability, resulting in facile interaction with cellulose and overcome the difficulty of solid-solid reaction. The monodispersed nanoparticles in form of fluid solution are able to promote large number of surface active site for more access to the oxygen atom in the ether linkage of cellulose.
- Magnetic properties acid catalyst: magnetization attraction with external magnetic field will facilitate the separation process and catalyst recovery. Presence of repulsion and attraction effect from magnetic nanoparticle is able to reduce the reaction barrier due to adsorption, agglomeration, dispersion, and viscous effects of the reaction mixture.
- Catalyst with homogeneous, biphasic, and heterogeneous system: catalyst will form liquid-solid phase during hydrolysis reaction through polar or nonpolar solvent and heating. It can be soluble in water and, therefore, more efficient for hydrolysis of solid cellulose. Furthermore, this catalyst can be recovered in solid form after reaction by distilling out the products and solvent.

### 6.1. Iron-Based Catalyst

Iron metal-based catalyst with its Lewis acid site can perform as catalyst for acid hydrolysis. Several studies reported that transition metal salts could increase hydrolysis rate for biomass fractionation and nanocellulose synthesis. Iron-based inorganic salts (FeCl_3_ and Fe_2_(SO_4_)_3_) had been selected as catalyst to accelerate the degradation of hemicellulose in corn stover. FeCl_3_ significantly increased the hemicellulose degradation in aqueous solutions heated between 140 and 200°C with high xylose recovery and low cellulose removal, amounting to ~90% and <10%, respectively. In this study, hemicellulose removal increased 11-fold when the corn stover was pretreated with 0.1 M FeCl_3_ compared to pretreatment with hot water under the same conditions, which was also 6-fold greater than pretreatment with dilute sulfuric acid at the same pH [56]. This finding was supported by Liu's group [57], where FeCl_3_ significantly increases the hemicellulose (xylose and xylotriose) degradation at 180°C. The result showed that 0.8% of FeCl_3_ with acid pH of 1.86 rendered 3-fold and 7-fold greater of xylose and xylotriose degradation, respectively, than that for treatment with dilute sulfuric acid with similar pH value. This suggested that different mechanism may exist for the reaction; first, the presence of FeCl_3_ may induce the formation of H^+^ ion from water for hydrolysis; second, FeCl_3_ may catalyze the dehydration of carbohydrates. FeSO_4_ catalyst also showed strong effects on the pretreatment of corn stover, where hemicellulose significantly degrades to xylose (60.3%) with high recover of cellulose (89.8%) under temperature of 180°C within 20 min. According to Zhao et al. [58], FeSO_4_ pretreatment is capable of disrupting the ester linkages between cellulose and hemicellulose. It is a potential pretreatment that is able to enhance further hydrolysis reaction of lignocellulosic biomass by destructing chemical composition and altering structural features. In acid hydrolysis, proton (H^+^ from HCl or H_2_SO_4_) plays an important role to weaken the glycosidic bond energy by attracting electron, making it easier for bone rupture. Metal ions also play same function as proton, which consist of more positive charge to pair with more electrons, but proton can only pair with one electron. This fact was in agreement with the studies above, where FeCl_3_ (trivalent, Fe^3+^) rendered better hydrolysis effect than FeSO_4_ (divalent, Fe^2+^), while acid solution performed less effect as compared to inorganic salts [113].

Recently, researchers started to use transition metal-based catalyst (FeCl_3_) for preparing cellulose nanocrystal (CNC) via hydrolysis of cellulose. The results show that 22% of CNC was produced under 10% FeCl_3_, temperature of 110°C for 60 min, and ultrasonic time of 180 min. CNC produced are shaped rod-like with the diameter of 20–50 nm, the length of 200–300 nm, and crystallinity of 76.2% [59].

Other than iron salts, the mesoporous structure exhibited in iron oxide catalyst allows accessibility of feedstock to active acid sites inside the pores. The characteristics of mesoporous iron oxide for cellulose hydrolysis are [114]

- high surface area;
- tunable pore size to improve accessibility of reactant;
- good thermal stability;
- good acidity;
- nanosize for better dispersion and active sites accessibility.

Paramagnetic-based nanocatalysts have attracted much interest due to their unique properties. The acidity of this nanoiron catalysts suspension (Zn–Ca–Fe based catalyst), capable to tune by adjusting the composition of Fe content, resulted in high accessibility of catalytic active sites to hydrolyse glycosidic bond in cellulose [60]. With the presence of Fe in the catalyst system, the catalyst is capable of physically separating from end product by applying external magnetic field [107]. So far, this catalyst was used as the hydrolysis catalyst for depolymerisation of cellulose into glucose monomer.

Same case goes to Fe_3_O_4_, although it consists of potential magnetization properties suitable for separation process for most of the catalytic systems; however, it is so far applied in glucose synthesis [115]. Generally, Fe_3_O_4_ nanoparticles will incorporate with acid carrier such as carbonaceous support (Fe_3_O_4_@C–SO_3_H) [61] and mesoporous silica support (Fe_3_O_4_-SBA-SO_3_H) [62] as an acid catalyst while enhancing efficient catalyst recovery system. Zhang et al. [61] reported that the Fe_3_O_4_@C–SO_3_H nanoparticle is composed of magnetic Fe_3_O_4_ that doped in a sulphonated carbon shell, which render good characteristic in terms of magnetization separation effect, high stability, and good reusability. The acid catalyst showed 48.6% cellulose conversion with 52.1% glucose selectivity under the moderate conditions of 140°C after 12 h reaction. Experiments on the hydrolysis of cellobiose with Fe_3_O_4_-SBA-SO_3_H showed that the magnetic solid acid gave an even better performance than sulfuric acid as well [62]. Thus, supported Fe_3_O_4_ nanocatalyst provides good access of macrocellulose to SO_3_H group with its functional characteristic which allows it to be separated and regenerated after the process.

### 6.2. Heteropoly Acid

Heteropoly acids, HPA (e.g., H_3_PWO_12_O_40_) with its unique physicochemical properties, Bronsted acidity, stability, and high proton mobility, have potential to be used for cellulose hydrolysis. It can be tuned to homogeneous, biphasic, and heterogeneous system at different conditions. HPAs are soluble in water and possess acidic strength similar to sulfuric acid. In theory, HPA acted as H_2_SO_4_ by donating H^+^ ions to oxygen atom in the glycosidic ether bonds of cellulose for depolymerisation process. HPA performed as homogeneous catalyst during hydrothermal hydrolysis, and it can be recovered to solid phase by employing organic solvent such as diethyl ether [63, 107, 116, 117].

The HPA catalyst system in cellulose hydrolysis involved three catalytic modes [118]: (i) surface reaction; (ii) pseudoliquid (bulk-type 1); and (iii) solid bulk-type II. Macrosize cellulose normally hydrolyses on the surface of solid HPA catalyst to reduce the crystallinity of cellulose structure. The smaller molecule of cellulose will later diffuse into the solid catalyst and undergoes reaction with pseudoliquid phase followed by solid bulk-type II diffusion of electrons and protons to assist the redox surface.

Jiang's study showed that a remarkably high yield of glucose (50.5%) was achieved via acid hydrolysis of H_3_PW_12_O_40_ under reaction conditions of 180°C and 2 h of reaction time [63]. Furthermore, Cs_*x*_H_3−*x*_PW_12_O_40_ (Cs-doped HPA) has gained much attention for selective hydrolysis of microcrystalline cellulose (MCC) to sugars in the aqueous phase. Partial substitution of proton by Cs^+^ improves the characteristic of HPA, where the surface acidity increases with higher surface area, controlled shape selectivity and good hydrophobility effect (insoluble in water). According to Tian's study, Cs_1_H_2_PW_12_O_40_, with the strongest protonic acid site, showed the best catalytic performance in the conversion of MCC for acid hydrolysis, where highest total reducing sugar (TRS) and glucose yields were 30.1 and 27.2%, respectively [64].

Most of the studies used HPA-based catalyst for cellulose hydrolysis to glucose, which was further processed to bioethanol for biofuel application. A control mechanism has to be explored to degrade macromolecule of cellulose to nanomolecular size by using HPA catalyst. It has been suggested that lower reaction temperature and shorter reaction time are the best choice to avoid overdegradation of cellulose to glucose monomer in HPA catalyzed reaction.

### 6.3. Ion-Exchange Resin

Ion-exchange resin commercially acted as solid acid catalysts in reactions such as alkylation with olefins, alkyl halides, alkyl esters, isomerization, transalkylation, nitration, and hydrolysis. Compared with homogeneous liquid, ion-exchange resin is easily separated after reaction, less water washing, low equipment corrosion, and high reusability. Huang Biao's team uses cation-exchange resin as an alternative to liquid acid for the hydrolysis of microcrystalline cellulose (MCC) into nanocrystalline cellulose (NCC) with the aid of ultrasonification treatment. This novel hydrolysis showed that 50.04% of NCC (2–24 nm) was achieved at a ratio of resin: MCC (w/w) of 10, temperature of 48°C, and reaction time of 189 min. The crystallinity of hydrolysed MCC was found to be increased from 75.2% to 84.26%, which indicated the removal of amorphous region and realignment of cellulose molecules [65].

## 7. Conclusion

Lignocellulosic biomass is the most abundant and biorenewable polymer on earth with great potential for sustainable nanocellulose production. The efficient and controlled breakdown of natural cellulose would produce nanocellulose, which is a mother compound for the synthesis of a large number of chemicals for food, energy, advance material, health, and environmental applications. Nanocellulose can be used as an immobilization support for chemical, microbial, and enzyme-catalysts. Synthesis of synthetic rubbers, bioplastic, pharmaceuticals materials, methyltetrahydrofurans, butanediol, and lactones from nanocellulose would be a quantum leap in nanocellulose industry. Nanocellulose and its derivatives could be further used to synthesize conducting polymers which could be used in biosensor appliances as well as molecular sieves. The complex hierarchy structure of lignocellulose is the main obstacle for major components separation. Overcoming the recalcitrance of lignocellulosic biomass is a key step in separating the biopolymer. Existence of lignin and the stability induced by inter- and intramolecular hydrogen bonding of cellulosic materials makes it a challenge for catalyst design. The current methods to convert cellulose into nanocellulose are based on acid, alkali, supercritical water, and thermal hydrolysis which often destroy the hierarchical structure of cellulose microfibrils and subsequently introduce impurities to the final products and produces unwanted by-products. The current methods also consume a lot of energy during and after the process and thus are deemed to be unprofitable and nonenvironmentally friendly. The typically used liquid-based catalysts are mineral acids and alkali. Introduction of novel catalysts such as iron-based catalyst, heteropoly acids, and ion exchange resin offer an integrated approach combining physical-chemical catalysts for the controlled structured degradation of natural cellulose into nanocellulose which promote more reliable methods in degradation of cellulose into nanocellulose. This shall further encourage more researches towards nanocellulosic field as more nanocellulose is readily available for utilization as a result of more efficient cellulose degradation process.

## Figures and Tables

**Table 1:** Chemical composition of common agricultural residues and wastes.

Types of biomass	Lignocellulosic substrate	Cellulose (%)	Hemicellulose (%)	Lignin (%)
Agriculture waste	Corncobs	45	35	15
	Wheat straw	30	50	15
	Barley straw	33–40	20–35	8–17
	Corn stover	39–42	22–28	18–22
	Nut shells	25–30	25–30	30–40

Energy crops	Empty fruit bunch	41	24	21.2
	Switch grass	45	31.4	12

Forestry waste	Hardwood stems	40–55	24–40	18–25
	Softwood stems	45–50	25–30	25–35
	Leaves	15–20	80–85	0

Industrial waste	Waste papers from chemical pulps	60–70	10–20	5–10
	Organic compound from wastewater solid	8–15	0	0

**Table 2:** Degree of biomass degradation by using various types of chemical treatments.

Chemical pretreatment	Type of biomass	Concentration	Ratio of BM : chemical	Temperature (°C)	Time (h)	C^a^	H^b^	L^c^	Monomeric sugar
Acid hydrolysis									
(i) Dilute acid									
Phosphoric acid [25]	Rapeseed	0.32% (w/w)	12 : 100	202	0.08	++	++	+	Recovered from hydrolysis of hemicellulose and cellulose
Sulphuric acid [26, 27]	Switch grass	1.2% (w/w)	3 : 100	160	4.3	++	++	+	Recovered from hydrolysis of hemicellulose and cellulose
(ii) Concentrated acid									
Sulphuric acid [28]	Bamboo	75% (w/w)	2.4 : 4.4	59	0.5	++	++	+	Saccharification of BM
Sulphuric acid [29]	Corn stover	65–80% (w/w)	1 : 20	121	1	++	++	+	Saccharification of BM

Alkaline hydrolysis									
Sodium hydroxide [30]	Wheat plant	8% (w/v)	5 : 95	75	1	+	+	++	Recovered from hydrolysis of hemicellulose and cellulose
Calcium hydroxide [31]	Switch grass	0.1 g	1 : 04	121	0.5	+	+	++	Recovered from hydrolysis of hemicellulose and cellulose

Organosolv									
Ethyl acetate, ethanol-water [32]	Prairie cordgrass, corn stover, and switch grass	ND	1 : 10	140	0.33	−	−	++	Limited degradation of sugar

Ionic liquid									
1-Ethyl-3-methylimidazolium acetate ([C2mim][OAc]) [26]	Switch grass	9.7 g	0.3 : 0.97	160	3	+	+	++	Recovered from hydrolysis of cellulose
1-Butyl-3-methylimidazolium chloride (BMIMCl) [33]	Sugarcane bagasse	ND	1 : 10	130	0.5	+	+	++	Recovered from hydrolysis of cellulose

Oxidative delignification									
Peracetic acid [34]	Cotton	40 ml/L	1 : 30	30–70	0.25–4	++	+	++	Recovered from hydrolysis of hemicellulose and cellulose
Peracetic Acid [35]	Aspen Wood	115 mM	1.3 : 40	60	6	++	+	++	Recovered from hydrolysis of hemicellulose and cellulose

++: hydrolysis towards cellulose, hydrolysis towards hemicellulose, and efficient removal of lignin;

+: less effect towards cellulose hydrolysis, less effect and removal of hemicellulose, and solubilisation of lignin;

−: minor effect toward cellulose, minor effect towards hemicellulose, and less efficient in removal of lignin;

BM: biomass.

^a^Cellulose.

^b^Hemicellulose.

^c^Lignin.

**Table 3:** Functionality, advantages, and limitations for each chemical treatments.

Chemical	Mode of action	Advantages	Disadvantages/limitation	Remarks
Dilute acid				
Sulfuric acid, phosphoric acid [36]	(1) Removal of hemicellulose	(1) Higher reaction rates (2) Increase the accessibility of cellulose	(1) Form by-product (fermentation inhibitors) (2) High cost and expensive construction material due to acidic environment (3) Corrosive to reactor	Minimal degradation of lignin and cellulose

Concentrated acid				
Sulfuric acid, phosphoric acid [37]	(1) Solubilisation of hemicellulose and direct hydrolysis of cellulose to glucose	(1) Suitable to all types of biomass	(1) Uncontrolled hydrolysis process (2) Corrosive to reactor	Suitable for the glucose synthesis (saccharification of biomass)

Alkaline hydrolysis				
Sodium hydroxide, calcium hydroxide [38, 39]	(1) Removal of lignin (major) (2) Removal of hemicellulose (3) Cellulose swelling	(1) High solubilisation of lignin (2) Low formation fermentation inhibitors	(1) High cost of chemical (2) Alteration of lignin structure	Suitable to use prior to direct fermentation of carbohydrates

Organosolv				
Mixture of organic solvent and water [40]	(1) Extraction of lignin (2) Complete solubilisation of hemicellulose	(1) High recovery of lignin (2) Organic solvent used can be recycled and reused (3) No grinding/milling of biomass feedstock (4) Selective pretreatment method for lignin extraction	(1) High cost of solvent (2) High energy consumption during solvent recovering process	Suitable for lignin fractionation process where high content of lignin can be recover for specialty chemical synthesis

Ionic liquid				
Imidazolium salts [41]	(1) Extraction of lignin (2) Decrease the cellulose crystallinity index (3) Carbohydrate dissolution	(1) IL is high thermal stability and low volatility	(1) High cost of chemicals.	The effects towards hemicellulose and lignin are depending on the nature of ionic liquid used

Oxidative delignification				
Hydrogen peroxide [42]	(1) Solubilisation of lignin and hemicellulose. (2) Bleaching effect to the pulp	(1) Efficient in removal of lignin (2) Increase biomass digestibility	(1) High costs of chemicals	Suitable for cellulose bleaching where lignin and hemicellulose will degrade in the presence of alkali

**Table 4:** Summary of depolymerisation treatments for nanocellulose synthesis.

Chemical treatment								
Chemical	Cellulose source	Concentration of chemical	Ratio of substrate : acid	Reaction time	Temperature (°C)	Pretreatment	Particle size (nm)∗	Yield of NC
Sulfuric acid [43]	Branch barks of mulberry (*Morus alba* L.)	64% (w/w)	1 : 10	0.5 h	60	Pulping treatment	20–40	N/A
Sulfuric acid [44]	Sugarcane bagasse	60% (w/v)	1 : 20	5 h	50	Pulping treatment	35	N/A
Sulfuric acid [45]	Waste cotton fabrics	63.5% (w/w)	1 : 15	3 h	44	Pulping treatment	20–100	21.50%
TEMPO mediated oxidation [46]	Pure rice straw	0.016 g	1 : 10	N/A	N/A	Oxidation	1.73	20%
Sulfuric acid [47]	Raw cotton linter	60% (w/w)	1 : 20	1 h	45	Grinding	12	N/A

Mechanical treatment								
Method	Cellulose source	Pressure/energy	Temperature (°C)		Pretreatment		Particle size (nm)∗	Yield of NC

High pressure homogenizer [48]	Wood pulp	500 bar	55–60		Pulping		ND	N/A
Sonification [49]	Wood powder from poplar trees	400–1200 W	<10		Pulping		5–20	N/A
High pressure homogenizer [50]	Sugarcane bagasse	40–140 MPa	130		Bleaching and ionic liquid treatment		20–100	N/A
Sonification [51]	Eucalyptus kraft pulp	50% amplitude, ~80 W	N/A		Pulping treatment		30	N/A
High shear homogenizer [52]	Bleached softwood pulp	22 000 rpm	N/A		Pulping and bleaching		16–28	N/A
High pressure homogenizer [53]	Nonwoody plants (flax, hemp, jute, and sisal)	600 bar	60–70		TEMPO-mediated oxidation		20–50	N/A

Biological treatment								
Enzyme/microorganism	Cellulose source		Time and temperature		Pretreatment		Particle size (nm)∗	Yield of NC

Anaerobic microbial [54]	Cotton fibers		7 days and 35°C		Pulping treatment		43 ± 13 & 119 ± 9	12.30%
Hemicell/pectinase and endoglucanase [55]	Curaua and sugarcane bagasse fibers		3 days and 50°C		Pulping treatment		55 ± 21	N/A

∗Measured by Transmission Electron Microscope (TEM).

N/A is not available; NC is nanocellulose.

**Table 5:** Factors that limit hydrolytic efficiency of cellulase on cellulose surface.

Factor 1 Biomass structural features	Factor 2 Enzyme mechanism
(I) Physical structure Accessible surface area, crystallinity, physical distribution of lignin in the biomass matrix, degree of polymerization, pore volume, and biomass particle size (II) Chemical structure Compositions of cellulose, hemicellulose (xylan), lignin, and acetyl groups bound to hemicellulose	(I) Enzyme diffusion and accessibility (II) Selectivity of enzyme to component in cellulose fiber (III) Enzyme inhibitor

**Table 6:** Solid acid catalyzed hydrolysis for nanocellulose production.

Feedstock	Catalyst	Acidity	Operation condition	Final product	References
Iron-based catalyst					
Corn stover	FeCl_3_	pH 1.68	0.1 M FeCl_3_, 140°C, 20 min	91% removal hemicellulose 91% recovery of cellulose	[56]
Hemicellulose compounds: xylose and xylotriose	FeCl_3_	pH 1.86	0.8% of FeCl_3_, 180°C, 20 min	65% degrade of xylose, 78% degrade of xylotriose	[57]
Corn stover	FeSO_4_	N/A	0.1 mol/L FeSO_4_, 180°C, 20 min	60.3% degrade of hemicellulose, 89.8% recovery of cellulose	[58]
Cellulose	FeCl_3_	N/A	10% FeCl_3_, 110°C, 60 min of reaction time, 180 min of ultrasonic time	22% CNC Diameter = 20–50 nm, Length of 200–300 nm, crystallinity index = 76.2%	[59]
Cellulose	Zn-Ca-Fe based nanocatalyst	N/A	160°C, 12 h	29% of glucose	[60]
MCC	Fe_3_O_4_@C-SO_3_H	1.30 mmol/g	140°C, 12 h 10 mL of water	52.1% of glucose	[61]
Cellobiose	Fe_3_O_4_-SBA-SO_3_H	1.09 mmol/g	120°C, 1 h,	98% of glucose	[62]

Heteropoly acid (HPA) catalyst					
Cellulose	HPA	N/A	180°C, 2 h	51% of glucose	[63]
MCC	CS-HPA	N/A	160°C, 6 h	30.1% of total reducing sugar (TRS), 27.2% glucose	[64]

Cation-exchange resin					
MCC	Cation-exchange Resin (NKC-9 cation-exchange resin (NKC-9))	Exchange capacity (mmol/g [H^+^]) ≥ 4.7, pearl size of 0.45–1.25 mm, and true wet density of 1.20–1.30 g/mL	Resin : MCC = 10 : 1, 48°C, 189 min under sonication	Yield of NCC is 50.04% Crystallinity index = 84.26%	[65]
